# Viral tRNA Mimicry from a Biocommunicative Perspective

**DOI:** 10.3389/fmicb.2017.02395

**Published:** 2017-12-05

**Authors:** Ascensión Ariza-Mateos, Jordi Gómez

**Affiliations:** ^1^Laboratory of RNA Archaeology, Instituto de Parasitología y Biomedicina “López Neyra” (Consejo Superior de Investigaciones Científicas), Granada, Spain; ^2^Centro de Biología Molecular “Severo Ochoa” (CSIC-UAM), Consejo Superior de Investigaciones Científicas (CSIC), Campus de Cantoblanco, Madrid, Spain; ^3^Centro de Investigación Biomédica en Red de Enfermedades Hepáticas y Digestivas (CIBERehd), Madrid, Spain

**Keywords:** tRNA-mimics, ceRNA, biosemiotics, biocommunication, RNase P, code, HCV, cetRNA

## Abstract

RNA viruses have very small genomes which limits the functions they can encode. One of the strategies employed by these viruses is to mimic key factors of the host cell so they can take advantage of the interactions and activities these factors typically participate in. The viral RNA genome itself was first observed to mimic cellular tRNA over 40 years ago. Since then researchers have confirmed that distinct families of RNA viruses are accessible to a battery of cellular factors involved in tRNA-related activities. Recently, potential tRNA-like structures have been detected within the sequences of a 100 mRNAs taken from human cells, one of these being the host defense interferon-alpha mRNA; these are then additional to the examples found in bacterial and yeast mRNAs. The mimetic relationship between tRNA, cellular mRNA, and viral RNA is the central focus of two considerations described below. These are subsequently used as a preface for a final hypothesis drawing on concepts relating to mimicry from the social sciences and humanities, such as power relations and creativity. Firstly, the presence of tRNA-like structures in mRNAs indicates that the viral tRNA-like signal could be mimicking tRNA-like elements that are contextualized by the specific carrier mRNAs, rather than, or in addition to, the tRNA itself, which would significantly increase the number of potential semiotic relations mediated by the viral signals. Secondly, and in particular, mimicking a host defense mRNA could be considered a potential new viral strategy for survival. Finally, we propose that mRNA’s mimicry of tRNA could be indicative of an ancestral intracellular conflict in which species of mRNAs invaded the cell, but from within. As the meaning of the mimetic signal depends on the context, in this case, the conflict that arises when the viral signal enters the cell can change the meaning of the mRNAs’ internal tRNA-like signals, from their current significance to that they had in the distant past.

## Introduction

**FIGURE 1:**
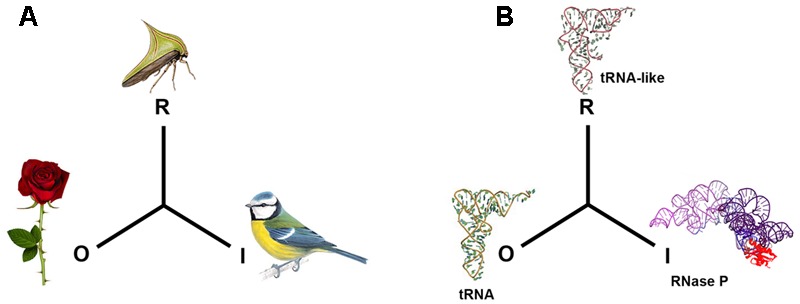
Mimetic relationships. In biosemiotic terms and according to the sign system of Peirce, the mimetic signal corresponds to the representamen (R), the model to the object (O) and the operator to the Interpretant (I) (Kilstrup, 2015). In **(A)**, the plant’s thorn is the Object (O), the thorn bug is the Representamen (R) and the predatory bird that the prey eludes is the Interpretant (I). In **(B)**, a molecular example: the tRNA^Phe^ is considered to be the Object (O), the 3′ untranslated end of the genome of plant virus TYMV is the representamen (R), while the interpretant (I) is the tRNA precursor processing enzyme RNase P.

In general terms, a mimetic system involves the interaction of three agents: the mimic that simulates the signals or features of a second agent, referred to as the model, in order to confuse a third entity, the operator. This confusion is in some way beneficial for the mimic (Pasteur, 1995). Mimetic similarities are only considered to be those that occur where the carrying agents have no common origin. The most widespread line of enquiry related to mimicry is based on Darwinian evolution, according to which, mimicry comes about through selective forces that favor false recognition, although this not always so, for example in a case where the variety of possible forms is limited (Maran, 2017). Other research emphasizes the communicative aspect: for Wolfgang Wicker, mimicry is based on the relationship of superficial similarity between organisms, and this relationship enables a flow of information (Wickler, 1998). This concept is rectified by Timo Maran, who places the emphasis on the signal. The important thing for this latter author is the similarity between the messages or signals emitted by the distinct organisms (which normally belong to different species); it is this signal that has mimetic value (Maran, 2017). This interpretative view of mimicry focuses less on the evolution of the mimicry itself and more on the different roles played by the various actors in the mimetic signal in an ecological context **Figure 1A**.

Mimicry was first studied in the 19th century, with research into butterflies and other organisms, but by the second half of the 20th century it had reached the molecular realm (Maran, 2017). A considerable number of biological activities have been described when it comes to molecular mimicry, many of which play important roles in viral reproduction, viral pathogenesis, and autoimmunity (Murphy, 2001; Alcamí, 2003; Christen et al., 2010; Drayman et al., 2013; Kropp et al., 2014; Oldstone, 2014). In viruses, many mimetic activities are achieved thanks to the structural and functional similarity between viral and cellular proteins. This class of mimicry is strategically valuable to a virus as it helps it to evade action by the host’s immune system. Various viral products have been described that imitate different defensive factors, such as cytokine cell receptors (Murphy, 2001; Alcamí, 2003), and even factors that are not directly related to antiviral defense, such as the mimicry of protein histones that end up influencing the antiviral gene expression (Marazzi et al., 2012). Another important field of study is viral mimicry based on RNA, with particular emphasis on the structural mimicry of the transfer RNA molecule (tRNA). Viruses from very diverse families and genera adopt tRNA-like structures in their genomes, through which they acquire a wide range of molecular cell functions (Springer et al., 1998).

## Historical Framework

At the beginning of the 1970s, biologists already knew about the fundamental aspects of the tRNA molecule structure and the way it participates in a code, which we know as the genetic code, as it is the adaptor that operates just at the interface between the polynucleotide sequence and the amino acid sequence. In addition, the complete sequence of tRNA^Ala^ had been elucidated, its clover leaf secondary structure was known (Holley et al., 1965), and there was a three-dimensional model in the form of a boomerang (Witz, 2003), very similar to the L-shape that would be determined years later and which is the standard form of tRNA (Kim et al., 1973). The code was accepted by the scientific community, but only as a metaphor, under the assumption that it would eventually be reduced to a set of favored interactions between the amino acids and their corresponding tRNAs. Surprising, as highlighted by Barbieri, until only a few years ago the genetic code would have been the only biological code that would have been present throughout the history of life on Earth. This had to be so, because the concept of a code is related to the correspondence between signals based on convention and that was something thematically alien to physics and chemistry; unless the codes were considered a way of restricting the different possibilities of the information (Barbieri, 2009). In that case, dealing with the information from the molecular signals represented by nitrogenous bases, which distinguish nucleotides from one another, whether taken one by one, in the form of a triplet in the case of the codons, or longer sequences that constitute the genes, became possible with the Probabilistic Theory of Information (Shannon and Weaver, 1949; Brillouin, 1968). In this theory, the signs are reduced to *bits*, in other words, certain quantities of information devoid of qualitative meaning, which represent a probabilistic value. This theoretical framework has facilitated some work in virology, such as the possibility of describing and comparing the quantity of information contained in viral populations (Wolinsky et al., 1996; Cabot et al., 2000) and evaluating the divergence between viral sequences in the course of evolution (Pan and Deem, 2011). Nevertheless, it should be noted that Information Theory, as a probabilistic theory, is only possible in a closed information space where it is possible to anticipate all the possibilities of this information, equivalent to predicting all the events that could occur or processes that could be carried out. Within this mathematical framework, and clearly influenced by Chomsky (Searls, 2002), Manfred Eigen searched in the rules of the molecular language (syntax) for the element to which all genetic information could be reduced. The shortcomings of these approaches are described in Hoffmeyer and Emmeche (1991) and Witzany (1995).

Some authors have proposed that the responsibility for this treatment of biological data lies in the context in which Information Theory flourished, i.e., World War II, as reviewed in Hoffmeyer and Emmeche (1991). The way information is dealt with in this theory may be completely valid in a space of military communication saturated with power relations (Foucault, 2001) where interpretation is non-existent, the language being that of a disciplined soldier or automaton. It is also likely that it has something to do with biology’s inferiority complex with respect to physics and chemistry, so that biology, in its longing to be perceived as a science of objectivity equal to that of physics and chemistry (Mayr, 1982), has surrounded the question of molecular language with mathematical theory. Recent science has also been strongly influenced by science philosophy, principally logic and mathematics (Witzany, 2010). This has been and continues to be the dominant trend, which has most recently been extended with systems biology.

Influenced by this prejudice, and in this context, the discovery of the genetic code did not lead the way to the study of communication and cell control based on a real molecular language (Witzany and Baluska, 2012). The subject of tRNA mimicry has been developed within this restrictive framework, as has the molecular biology of nucleic acids. Nevertheless, some authors and schools maintain that the reductionist and quantitative treatment of the information is not sufficiently explanatory for molecular biology. These authors, from different perspectives, have proposed: understanding the cell as a duality of digital and analog codes (Hoffmeyer and Emmeche, 1991); that information always is interpretation (Hoffmeyer and Emmeche, 1991); that at the molecular and cellular level there are more codes than just the genetic code (Barbieri, 2008); that even this latter has not been able to codify to itself (Witzany and Baluska, 2012), but requires the participation of a multiplicity of molecular agents; with conflictive and collaborative social relationships at the molecular level (Gómez and Cacho, 2001; Villarreal, 2009, 2015; Villarreal and Witzany, 2013; Witzany, 2014). In short, it is the new areas of knowledge that are interested, firstly, in content communication via the interpretation of signals and molecular codes, an area known as biosemiotics, and secondly, in the fact that this interpretation is context dependent (pragmatic) and classified as biocommunication (Witzany, 2010). We must emphasize that in the latter, the context refers not only to environmental characteristics but also the history of the factors that participate in the interpretation, their identity and particularly their social relationships.

There are some excellent reviews of viral tRNA mimicry in the literature that include the most significant results and interpretations in the field (Giegé et al., 1993, 1998; Springer et al., 1998; Giegé, 2008; Dreher, 2009, 2010). Here we outline results found in the classic works on tRNA mimicry that could be compatible with the biocommunication theory. They include work where the identification of tRNA-like structures has been made directly using tRNA metabolism enzymes, and defines tRNA mimics in an operative way (Springer et al., 1998). We highlight some interpretations these results would have made possible, and also their fall into disuse due to the failure to study the biocommunicative facet in favor of the structural aspect; subsequently, we propose a hypothetical expansion of the subject in that direction.

## Tracing Viral tRNA Mimics Using tRNA Modifying Enzymes

### tRNA-Like Structures in the 3′ Region of Viral mRNAs

Despite the restrictive way biological information has generally been dealt with, as described above, according to which the genetic code could be treated as a machine code, largely due to the specificity of the aminoacyl-tRNA synthetases, the fortuitous discovery was made of the incorporation reaction of the valine amino acid into the 3′ position of the Turnip Yellow Mosaic Virus (TYMV) genomic RNA (Pinck et al., 1970). It was seen that cellular tRNA^val^ did not participate in this reaction, but it was the viral RNA in the 3′ region of the genome that was being modified (Pinck et al., 1970). This discovery turned out not to be exclusive to the tRNA^val^ and RNA of the TYMV, soon other examples were found among the family of aminoacil-tRNA synthetases, in particular those specific for histidine (Sela, 1972) and tyrosine (Hall et al., 1972) as modifier enzymes and other plant virus RNA substrates, such as Brome Mosaic Virus (BMV) (Hall et al., 1972) and Tobacco Mosaic Virus (TMV) (Sela, 1972). This first set of results shows that one of the enzyme families that is key to decoding the genetic code (known as the operative code) (Schimmel et al., 1993; Ribas de Pouplana and Schimmel, 2001), the aminoacyl-tRNA synthetases, can recognize as their own a substrate with a different sequence from the tRNA sequence. Specific and erroneous recognition of a signal is the paradox indicating that the number of molecular alternatives each aminoacyl-tRNA synthetase can recognize is not known. In other words, the “operative” code is open —it is not a machine code.

In a second study group, factors other than the aminoacyl-tRNA synthetases were discovered, capable of recognizing and modifying the 3′ end of the viral RNA. These factors include: the ACC-tRNA nucleotidyl-transferase (Litvak et al., 1970), responsible for incorporating adenine and cytosine (CCA sequence) at the 3′ end of the tRNA; the translation elongation factor in prokaryotes; and ribonuclease P (RNase P) activity (Prochiantz and Haenni, 1973; Guerrier-Takada et al., 1988), which is able to specifically process the tRNA precursor (pre-tRNA), cutting between two nucleotides that separate the leading 5′ region of pre-tRNA and mature tRNA (Robertson et al., 1972). This second set of results extends this specific and erroneous recognition of the 3′ region of the viral mRNA to a variety of very different cell translation enzymes and factors (Giegé, 2008). This second group of results also indicates that the forms these viral RNAs adopt in the 3′ region are communicative elements, as they reveal a great deal of indetermination and ambiguity, the significance of which necessitates their maturation by a contextualized interpreter.

In a third group of results, it was discovered that the previously mentioned enzymes are able to modify or interact with other elements of non-viral origin, related to the processing and control of gene expression in cellular mRNA. Examples are the leader sequence of the mRNA of the Threonyl-tRNA synthetase (ThrS) (Springer et al., 1989), the pre-mRNA of several aminoacyl-tRNA synthetases (Guo and Lambowitz, 1992), and the bacterial 10Sa RNA (Komine et al., 1994) [subsequently known as transfer-messenger RNA (tmRNA)]. These data could have at least suggested the possible existence of a context of intracellular tRNA-like signals and recognition factors in which viral tRNA-like signals competed.

Indeed, this entire set of results could have displaced the approach of tRNA-like motifs from a structural and syntactic analysis to a tRNA-like mediated real language, that is the characterization of sequences and structural rules that govern the assignment of aa into each tRNA-like motif to an approach that would have shown the overall contextual aspect that affects the interpretive agents and that definitively gives meaning to the signals (Witzany and Baluska, 2012). If we had continued systematically with this experimental identification of tRNA-like elements with cellular enzymes, such as those described above, we could have compiled a wider collection of correspondences between viral tRNA-like structures and cell recognition factors that would perhaps have enabled the outlining of other possible interrelated codes.

On the contrary, progress in experimental techniques for identifying new tRNA-like elements underwent a change that made this approach obsolete. After the 1980s, techniques for retrotranscribing RNA into cDNA, and subsequent DNA sequencing, largely replaced the direct sequencing of RNA by fingerprinting, a very delicate and painstaking technique (Branch et al., 1989). DNA sequencing became widely available in molecular biology labs, and structural prediction and 3D modeling software began to be developed (Dumas et al., 1987). This changed the way of looking for new tRNA-like motifs (Fechter et al., 2001). The first step was to determine the sequence of an RNA motif of interest and then to check *in silico* if the structure was indeed folded in the form of a clover leaf, and only then to experimentally test whether it really could be modified by a tRNA metabolic enzyme (Konarska and Sharp, 1990; Michel and Westhof, 1990; Pilipenko et al., 1992; Felden et al., 1994; Fechter et al., 2001; Lukavsky et al., 2003). Therefore, although in the 1970s the identification of a tRNA-like motif centered on its recognition through an enzyme of tRNA metabolism (Springer et al., 1989), the fundamental criterion became that of structural similarity according to a human observer and hence the emphasis shifted to the molecular architecture. The development of this new pathway involved an increasing understanding of the different intramolecular interactions of RNA and improved 3D models, as well as the determination of the structures using methods with ever greater resolution (Lukavsky et al., 2003; Boehringer et al., 2005). The objective was achieved with the determination of the TYMV tRNA-like structure using X-ray diffraction (Colussi et al., 2014). This trend represented a departure from the operative search of tRNA-mimic motifs, which would have brought us closer to the signal-interpreter relationship of the language. Structural studies of plant viral tRNA mimicry have identified in tRNA-likes the determinants for specific recognition by Aa-tRNA synthetases and other enzymes (a syntactic level), as well as confirming the similarity with standard tRNA. The next experimental step should have been to demonstrate that the aminoacylated tRNAs serve as Aa donors in the synthesis of viral proteins. In this way, the correlation between structural and functional similarity (semantic level) would have been expanded. This idea has persisted in the field for decades (Haenni et al., 1973; Barends et al., 2003), without success (Matsuda and Dreher, 2006, 2007). The idea would be pertinent if the information space that includes the genetic code were closed, but through the various examples we have looked at we can see the opposite, that the genetic code is one of the communicative possibilities of the factors that manage the information contained in tRNA and tRNA-like structures. This is not only true in the exceptional case of a viral infection, but also in operon regulation in a healthy cell.

### tRNA-Like Structures in the 5′ Region of Viral mRNAs

In 2002, more than 30 years after the identification of the tRNA-like motif in the 3′ region of the TYMV genome, it was discovered that human RNase P recognizes and specifically cuts the 5′ region of RNA in the hepatitis C virus (HCV) *in vitro*, a finding that was presented and discussed in relation to the tRNA mimicry of plant RNA viruses (Nadal et al., 2003). Later, it was also observed that this motif could be recognized by the RNase P ribozyme of the cyanobacteria *Synechocystis* sp. under high salinity conditions (Sabariegos et al., 2004). The presence of tRNA-like structures in the 5′ region of viral mRNA is generalized in animal pestiviruses that are phylogenetically related to HCV, such as bovine viral diarrhea virus (BVDV), and classical swine fever virus (CSFV) (Lyons and Robertson, 2003); as well as in the RNA of unrelated viruses, like the cricket paralysis virus (CrPV) (Lyons and Robertson, 2003), picornavirus food and mouth disease virus (Serrano et al., 2007), and polio virus (Andreev et al., 2012). Although there is no similarity in either the nucleotide sequence or secondary structure between the RNA in hepacivirus, picornavirus, and CrPV, these motifs are found in the 5′ region of coding sequences in these viruses, known as internal ribosome entry sites (IRES) (Lozano and Martínez-Salas, 2015). However, in HCV an additional motif was also identified between the region coding for structural and non-structural proteins (Nadal et al., 2003).

Thus, the identification the tRNA-like structure in the 5′ region of viral mRNAs began under the same operative definition as that used in the work to characterize the tRNA-like structure in the 3′ region of plant viruses. However, unlike the 3′ region of RNA, which can be examined by multiple specific and distinct enzymatic activities, the enzymatic identification of a tRNA-like structure located within a longer RNA strand is very limited. The possibilities are limited to RNase P, although in certain specific cases another endonuclease, RNase Z (Wilusz et al., 2008), and an enzyme that modifies tRNA nucleotides have been used successfully (Baumstark and Ahlquist, 2001).

But again, the pathway leading to the communicative aspect would be relatively blocked by two issues. The first relates to the fact that the subject is strongly influenced by the structuralist idea that it is necessary to determine the RNA structure before deciding whether this may be a tRNA mimetic structure. Therefore, cryo-electron microscopy led to the recognition of the L-shaped tertiary structure of the HCV IRES RNA (Boehringer et al., 2005) and the ribosome-bound CrPV (Fernandez et al., 2014). Subsequently, the resolution of the crystal structure of HCV RNA in the zone recognized by RNase P (Piron et al., 2005) indicated the presence of not a single, but rather a double pseudoknot (Berry et al., 2011). Without a doubt, this approach enables the mimetic resemblance to be visualized and partly explained, but it introduces the matter of the human observer (Maran, 2017). The second issue, which we detail below, relates to the specificity of the RNase P activity. It is a subject that requires particular attention and which we will look at below.

### RNase P Recognition Specificity

Various prestigious and influential groups in the field have assessed the specificity of RNase P and come to opposing conclusions. Whereas for some groups it represents a tool with therapeutic potential exactly because of its recognition specificity (Altman, 1995; Yuan and Altman, 1995), for others this represents non-specific activity because it can cut mRNAs in positions where bioinformatic folding indicates it is in a single strand form (Marvin et al., 2011).

Originally, and for many years, the only biological activity attributed to RNase P was the processing of pre-tRNA (Robertson et al., 1972; Altman, 1975; Jarrous and Reiner, 2007). Taking pre-tRNA as a model, the sequence and structure requirements of the RNA substrates of both *Escherichia coli* and human RNase P were carefully studied (Forster and Altman, 1990; Yuan and Altman, 1995; Liu and Altman, 1996; Werner et al., 1997, 1998). The cutting determinants for both endonucleases were found to be similar but not identical. For the RNase P of *E. coli* it is necessary for the T-stem loop next to the acceptor stem to terminate with the sequence –CCA (Liu and Altman, 1996), whereas for the human one, it is necessary to have a sequence of at least one nt between the T-stem and acceptor stem (Werner et al., 1997). Studies where the RNase P RNA domain is used as an antiviral agent against various cytomegalovirus mRNAs in culture, confirm that RNase P expressed in cytoplasm, besides acting as an antiviral agent, is not toxic to cells (Liu and Altman, 1995; Jiang et al., 2012).

Taken together, the results demonstrated that the RNase P was able to cut other substrates, different from tRNA but with which they shared structural similarities, like the RNA of the TYMV (Guerrier-Takada et al., 1988). Nevertheless, it was also observed that the RNase P was able to process substrates that were structurally different from tRNA, particularly when the ribozyme occurred in the presence of the protein subunit, as in the case of 4,5S RNA (Peck-Miller and Altman, 1991). This conferred to the protein the value of extending enzyme’s substrate recognition, allowing it to cleave single stranded RNA substrates as small as 5 nt (Hansen et al., 2001). In contrast, other results gave clues as to the requirements for substrate recognition by RNase P. One study found that RNase P processed the ribosomal RNA in yeast (Chamberlain et al., 1996); due to the similarity of the genomic organization of the rRNA precursors in yeast and *E. coli*, where the pre-tRNA are found in the same precursor molecule that contains the rRNA, a phylogenetic relationship explaining this and other cases could not be ruled out. In a completely different vein, there are studies where RNase P processes RNAs that can assume different configurations and whose transition is regulated by small metabolites (riboswitches) (Altman et al., 2005; Seif and Altman, 2008), in such a way that the substrate form is not the most thermodynamically stable (Altman et al., 2005). A systematic study in our laboratory has enabled us to evaluate the number of random mutations that are necessary to be introduced into a population of a standard RNA substrate for RNase P, RNase III and the binding to microRNA-122, and the binding of subunit 40S of the ribosome, and which halves these activities and interactions. It turns out that this number is similar for RNase P, the binding to 40S, and the binding to miR-122, and it is greater for RNase III. Therefore, if it is possible for RNase P to recognize multiple structures, its specificity is greater than that of RNase III and similar to that of the binding of a microRNA to its mRNA substrate and the binding of the HCV IRES to the 40S subunit of the ribosome (Prieto-Vega, 2016).

Based on the evidence presented above, we conclude that RNase P is no more non-specific than other factors for which we are confident of their specificity, and that if a substrate is processed by RNase P, the idea that there must be a structural similarity between this RNA motif and the tRNA, in the view of the human observer, is not correct.

## tRNA-Like Structures Inside Cellular mRNAs

Up to this point, tRNA mimicry been has limited to the interpretations of tRNA as a model (**Figure 1B**). A new study was undertaken to verify whether among the population of hepatic mRNA species there were any that were similar to the tRNA-like structure identified in the 5′ region of HCV RNA (Díaz-Toledano and Gómez, 2015). It was used as recognition factors human RNase P (Bartkiewicz et al., 1989) and the RNase P ribozyme of the cyanobacteria *Synechocystis* sp (Vioque, 1992; Pascual and Vioque, 1999). It was deduced that more than 100 species of mRNA were a substrate of the activity *in vitro*. Of these, three messengers were analyzed separately. These messengers were related to distinct historical periods of life: that of interferon alpha (IFNA), which originated in the vertebrates; that of histone H2A, related to the “DNA world”; and ribosomal protein S9 from the small ribosome subunit, related to the “RNA world.” It was observed that the three competed specifically with the pre-tRNA in a standard RNase P processing reaction, that the chemistry of the newly generated 3′OH and 5′PO_3_ ends confirmed it was RNase P, and that the minimum size fragment necessary to sustain the cleavage reaction was around a minimum of 120 nts, sufficient to house a tRNA-like structure. In addition, they were recognized by the *Synechocystis sp.* RNAse P ribozyme. The conclusion here should be that mRNAs with tRNA properties have been identified and characterized, thus expanding the repertoire of motifs that viral RNAs can mimic to host mRNAs. It has been proposed that each element may mimic more than one structure class of cellular substrate and may carry out more than one function (Alexander-Brett and Fremont, 2017). Thus, a viral tRNA-like mimicking a structure within an mRNA does not necessarily mean that it will not continue to mimic tRNA for other functions, but simply that it expands the number of functions which this structure may support.

**FIGURE 2:**
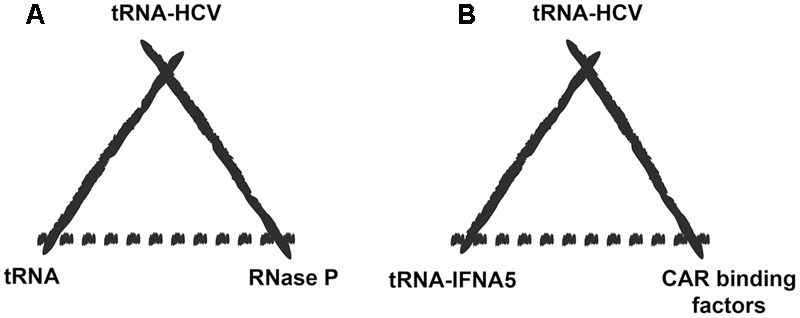
Extended biosemiotic relationships of tRNA mimics. **(A)** A particular example of tRNA mimicry would be the case of hepatitis C virus mRNA carrying a tRNA mimic element (representamen) that is specifically recognized by RNase P (the interpretant), which normally processes the tRNA precursor molecule (the model). **(B)** Potential switch in the tRNA mimetic relationship after the discovery of a tRNA-mimicking element in IFNA5 mRNA. The model for the viral RNA to mimic could now be a cellular mRNA rather than the tRNA molecule itself (in this case the mRNA for liver IFNA5). The deceived interpreter would be those factors that naturally interact with the IFNA5 mRNA-tRNA-mimicking region, such as CAR binding factors.

In biosemiotic terms, this supposes a paradigm change for the interpretation of the role of viral tRNA mimics due to the possibility that cellular mRNA species may become the “model” for the viral mRNA to mimic (Díaz-Toledano and Gómez, 2015) (**Figure 2**).

On the other hand, the mRNA that codes for the viperin protein, which is also related to antiviral defense, had been identified as a potential substrate for RNase MRP and RNase P (Mattijssen et al., 2011). Although the authors cannot discern which of the two RNases is responsible, this result could indicate the existence of a second family of antiviral defense mRNAs carrying tRNA-like elements.

### Structure and Possible Function of IFNA mRNA tRNA-Like Regions

**FIGURE 3:**
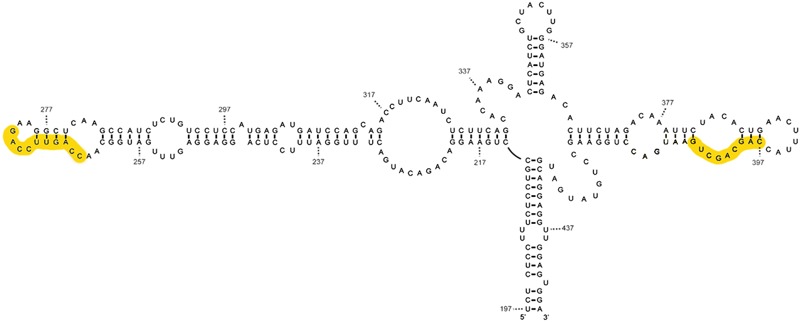
Mimetic structure of the tRNA-like of IFN5A. This image shows the secondary structure of the tRNA-type element of alpha interferon mRNA subtype 5 determined by various chemical and enzymatic methods. The CAR-E sequences are highlighted in yellow.

The proposed tRNA-like structure of IFNA mRNA is shown in (**Figure 3**). With respect to the recognition requirements of RNase P, domain 3 would represent the T-stem and T-loop of tRNA and domain 4 the acceptor stem (Díaz-Toledano and Gómez, 2015), without the need for the presence of the pseudoknots located around the helix junction for recognition by RNase P, as is characteristic of plant virus tRNAs. The pseudoknots in IFN5A might be responsible for the adequate orientation of the branches, as in the tRNA-like structure in the tobacco mosaic virus, where the helix junction is integrated within a pseudoknot (Felden et al., 1996).

The comparison of the tRNA-like structure in the 5′ region of HCV and IFNA mRNAs shows that there are remarkable similarities with regard to the polarity of the processing determinants, the sequence, the secondary structure, the presence of two pseudoknots and their location in the mRNA -described in Díaz-Toledano and Gómez (2015). The smallest fragment of IFNA 5required for RNase P processing (positions 242–424) coincides significantly with a region that is able to interact with nuclear export and cytoplasmic stabilization factors (TREX) (Lei et al., 2011). Functional CAR signals involve primary sequence elements (one to four) 10 nts in length, referred to as CAR-E (Lei et al., 2013). Each CAR-E is located at the extremities of domains II and IV of the IFNA tRNA-like motif, in positions 264–273, and 396–405, as depicted in the yellow boxes in **Figure 3**. Apart from this positional symmetry, CAR-E adopts an equivalent secondary structure.

This could be thought of as selective interference of HCV with the expression of IFNA-5 in the liver as a result of direct competition for factors eventually facilitating cytoplasmic accumulation of IFN5A mRNA (Díaz-Toledano and Gómez, 2015). In fact, the INFA signaling pathway is utilized in cell defense against HCV, although the IFNA5 liver-specific sub-type of IFNA mRNA disappears from the liver several days after infection (Castelruiz et al., 1999). Alternatively, or additionally, HCV RNA may benefit from using the cell factor TREX.

Finally, the detection of mimetic elements that are recognized by RNase P in cellular mRNAs, and in particular in IFNA mRNA, leads to a new paradigm. This provides a new way of looking at the function of a variety of viral tRNA-like motifs, as this type of structural mimicry might be related to specific host mRNA species rather than, or in addition to, tRNA itself, an issue which for the moment we can only deal with hypothetically.

## What Changes when tRNA-Like-mRNA is Used Instead of tRNA as a Model for Viral Mimesis?

### Evolutionary Context: Origin of tRNA-Like Structures in Cellular mRNAs

The origins of tRNA go back to the “RNA world” (Rodin et al., 2011; Di Giulio, 2012; Caetano-Anollés and Sun, 2014; Caetano-Anollés and Caetano-Anollés, 2016). In the case of tRNA-like structures in mRNAs, theoretical studies along with certain experimental results seem to insist on an idea first suggested by Eigen and Winkler-Oswatitsch (1981). Eigen asked himself if ancestral tRNA could have worked like mRNA in the RNA world. His idea was that both the populations of RNA capable of aminoacylating and functioning as adapters, such as those that act as messengers, could have been selected from a single population of replicative RNA quasispecies (Eigen and Biebricher, 1988; Domingo, 2007; Mas et al., 2010), positive and negative strands. He identified a series of requirements that would be necessary for this to occur: symmetry between positive and negative chains, a high number of G:C pairings, and small-sized RNAs, among other features, which would guarantee stability and structural complementarity between the two types of molecules. Since then, different groups have suggested and supported this hypothesis, based on different observations or proposing distinct models (Ohnishi et al., 2002, 2005; Bernhardt and Tate, 2010). The two most recent proposals are that either the original substrates of mRNA were aminoacylated proto-tRNA-like structures with regions of self-complementation (Di Giulio, 2015), or that they were aminoacylated proto-tRNA-like structures with the capacity to take on different configurations (de Farias et al., 2016). These original substrates would have been concatenated to form mRNA. An alternative, and also recent hypothesis is that a population of molecules that already contained tRNA-like and mRNA-like domains (proto-tmRNA) acted as a common ancestor of molecules that elongated and eventually evolved into tRNAs and mRNAs (Macé and Gillet, 2016).

A study by our lab, in which we evaluated the sensitivity of the human hepatic mRNA population to human RNase P, reveals interesting data relating to these proposals on the origin of mRNA. The competition results with Poly-rA, pre-tRNA, and human liver mRNA, in a standard pre-tRNA RNase P processing assay, indicate that the mRNA population competes well with the natural substrate processing of RNase P (Figures 1A–C) in Díaz-Toledano and Gómez (2015). At the molar level, it is six times better competitor than the pre-tRNA itself. The results from the competition are very similar to those obtained by Dr. Engelke’s group using RNase P and yeast mRNAs Figures 2B,C in Marvin et al. (2011), although that study did not provide data on the competition with the pre-tRNA. However, direct examination of the products from the mRNA population after incubation with human RNase P did not reflect processing, which was also in line with studies published in the 1970s that had tested the cleavage of hnRNA using RNase P-enriched extracts (Ferrari et al., 1980; Kole and Altman, 1981), and did not observe processing. When instead of examining the mRNA population, human or yeast mRNA species were tested separately (Marvin et al., 2011; Díaz-Toledano and Gómez, 2015), it was seen that these mRNAs were sensitive to being processed by RNase P in specific positions, but there was a very low cutting effectiveness. It is possible the internal structures of mRNA that are able to inhibit the enzyme in a competition test, are nevertheless not sufficiently stable or do not present an optimal configuration to be actively processed. These results could support the hypothesis of the origin of mRNA based on concatenations of tRNA-like molecules, where their structures have been undergoing alterations since their origin. Specifically, the presence of RNase P activity from the “RNA world” to the present would have posed a danger for the proto-tRNA-like polymer because it would have facilitated its fragmentation, making it a selective force that would have tended to alter the proto-tRNA-like structures integrated into the messengers, favoring an imperfect or unstable similarity to tRNA, rather than resembling it perfectly.

Alternative explanations for the origin of tRNA-like structures within mRNA are that these structures have evolved from other forms in the mRNAs, or have been contributed by viral agents that subsequently colonized the genes of the mRNAs at different stages of life, taking advantage of the similarity of some of the tRNA properties. However, if so, these properties do not seem to be related to the tRNAs involved in protein synthesis, firstly, because they are found within a longer molecule that prevents aminoacylation, and principally, because in the examples of plant virus RNA where the tRNA occupies the 3′ position and is able to aminoacylate, this does not act as an Aa donor. Thus, in any case, it returns to the ancestral properties of tRNA.

In summary, the RNase P competition tests reinforce the idea that current mRNAs could have originated from the concatenation of proto-tRNA-likes or the elongation of the original transfer-messenger RNA-type molecules (tmRNAs), which have degenerated, but which in some cases would still contain vestiges of the proto-tRNA-like originals.

### Environmental Context: tRNA-Like Structures in mRNAs

The presence of tRNA-like structural motifs in cellular mRNAs (tRNA-like-mRNA) extends the structural space of tRNA forms (Gilbert and Labuda, 1999; Wilusz et al., 2008). The molecules of the tRNA family are very similar to one other, they play a central role in the molecular biology of the cell, although they also participate in other pathways (Giegé, 2008; Maute et al., 2014; Katz et al., 2016), and at a quantitative level this is the most abundant type of molecule; in contrast, tRNA-like-mRNA have distinct sequences, are found in smaller proportions, and the mRNAs that carry them participate in very diverse functions.

## The Archeological Reconstruction of tRNA-Mimicry

### Communication in the World of RNA and tRNA-Like Structures

In the primordial world, certain forms of communication would have been increasingly necessary and complex as it was necessary to record the external and internal changes of a primitive cell, and to readjust the metabolic processes ever more precisely. This would have required signals, receptors, riboswitches (Nelson and Breaker, 2017), codes (Caetano-Anollés et al., 2013), and communicative networks that integrated and channelized the reception and response to those changes. The vehicles for these signals would have been small molecules derived from ribonucleotides (Nelson and Breaker, 2017) as well as from longer RNAs, such as stem-loops and proto-tRNA-likes (Witzany, 2014; Villarreal, 2015). This supposes a level of communication that had to follow a series of rules according to capacities and biochemical requirements, as well as the necessity to belong to some or other of the participatory consortiums (Witzany, 2017a). Some of these requirements could have been: (i) the Eigen limit (Eigen and Biebricher, 1988), as due to the high rate of mutation in primitive replication, the size a replicative RNA could maintain without losing information is small (50–100 nts); (ii) participation in a molecular consortium (Witzany, 2014; Villarreal, 2015), requiring the prior molecular recognition of itself which determines a language with a repetitive syntax (Witzany, 2017b); (iii) and finally, the relationship between sequence space and structural space (Schuster, 1997) would have degenerated, in such a way that multiple sequences would group into very few distinct structural folds, greatly favoring structural mimicry. All these requirements would favor an RNA world inhabited by mini-helices that either through duplication or accretion could have created an environment very rich in forms of proto-tRNA-likes (Tamura, 2011; Caetano-Anollés and Caetano-Anollés, 2016). These forms could have included the ancestors of both the aminoacylated tRNAs and the ribosomal peptidyl transferase center (de Farias et al., 2017) and the mRNA (de Farias et al., 2016).

The RNA world would have been the context in which those proto-tRNA-likes would have gone from being free to being embedded in mRNA during the concatenation process or via continuous growth, if we accept the hypotheses presented above. The concatenation would have probably followed the selection rules suggested for “conjoined RNAs” by Robertson and Neel (1999):*“in that two independent activities, each embodied in a separately evolved RNA are required to survive the conjunction or capture process that joins them together.”* In other words, in a primitive stage, the formation of each of the proto-mRNAs would have been selectively favored by the sum of the activities of the tRNA-like molecules that are bound together to form a longer RNA. These capabilities could be related to the interaction of factors that confer RNA stability, association with ribosomal subunits, sensitivity to conformational changes in the presence of small molecules, and so on. All these possibilities should have been conferred on the mRNAs with greater communicative capacity, therefore participating in increasingly complex sections in the original cell.

### tRNA-Like Structures in mRNAs after the Development of Protein Synthesis

After the complete development of protein synthesis using the mRNA as a template, a new language was incorporated into the cell, whose syntax was mainly non-repetitive, and in which the semantic function was separated from the sequence of RNA bases by being carried by another material support, the protein. The latter allows for post-translational modifications of the proteins while the mRNA remains intact, producing native proteins, or for the RNA to be edited in various ways. All these changes enriched the communication, to which must also be added the enzymatic activities of proteins that greatly expand their communicative ability. Meanwhile, the contextual dimension did not stop expanding, and it did so in a very elaborate way, where the signals became immersed in positive and negative feedback processes, cascade amplification, molecular reorganization, editing, vacuolization, agglomeration, molecular addiction, and so on.

With all this transformation and the continuous selective pressure to increase translation effectiveness, the majority of the communicative capacities that the tRNA-like molecules would have contributed to mRNAs when integrating into these would have disappeared, others would have become obsolete when their primitive receptors vanished during the extinction of ribozymes and RNA-world forms, and others would eventually have become controlled but not completely extinguished. There are examples that provide certain support to this non-total-elimination, both in viral and cellular RNAs.

At present, the interaction of cellular mRNAs and the ribosome during translation initiation is mediated by mRNA signals and a battery of protein factors that are very specific to that process, including translation initiation factors and other proteins with more varied roles (Jackson et al., 2010). To initiate the translation of the second ORF of CrPV none of this is required (Jan and Sarnow, 2002). To do this, the virus contains an internal ribosome entry site (IRES) that imitates tRNA with its anticodon bound to a messenger when this is present in the P site of the ribosome, enabling a tRNA^Ala^ to enter site A, and allowing translocation, even though the peptide bond has not formed (Spahn et al., 2004; Costantino et al., 2008). This happens both *in vitro* and in cells, although in this latter case it can also begin at the P site (Kamoshita et al., 2009).

Another example also related to the initiation of CrPV translation is the ability of the IRES of this eukaryotic virus to operate in the translation system of both eukaryotes and prokaryotes. The authors who determined the crystal structure of the CrPV IRES bound to the bacterial ribosome, state that the CrPV IRES bridges billions of years of divergent evolution by being recognized by two different domains of life (Colussi et al., 2015). This finding provides new evidence on a proposal from the 1970s that indicated that at least some signals present in the mRNA, leading to recognition by the ribosome, should be common to both prokaryotes and eukaryotes, despite the signal incompatibility between the two translation systems. In these studies, using ribosomes from reticulocyte lysate, it was observed that the authentic translation initiation sites of the bacteriophage f1 mRNA could be protected from RNase A (Legon et al., 1977). In addition, it had been determined that eukaryotic viruses, such as polioviruses, could function in translation extracts from a prokaryotic origin (Rekosh et al., 1970).

We would like to emphasize that there is another series of evidence also based on tRNA-like signals that currently function as part of the cellular mRNA. Such is the case of plants, where the long distance transport of mRNAs between distinct tissues seems to be determined by tRNA-like structures in mRNAs (Zhang et al., 2016). Or the case of IFNA, where the enormous amount of coincidence between the region forming the cloverleaf structure, and the region that signals for TREF binding seems to indicate that the tRNA-like signal plays a role in the transport of the nucleus to the cytoplasm and participates in mRNA stability (Lei et al., 2011, 2013; Díaz-Toledano and Gómez, 2015). Also in bacteria, it has been known for decades that the leader region of Threonyl-tRNA synthetase mRNA (ThrRS), whose structural similarity to the tRNA^Thr^ anticodon allows it to bind to the ThrS protein, reflects a functional activity in the expression control of this mRNA (Springer et al., 1998).

There is an additional example, which although not related to tRNA-like structures, does indicate that certain archaic RNA signals have not lost their primitive function (Jha et al., 2017). Normally in mammal cells, long poly-A sequences located upstream of the AUG initiator work against the translation, the opposite of what happens in translation in plants. Some poxviruses from mammal cells contain those poly-A stretches upstream of the AUG initiator and optimize protein synthesis in the infected cell, mimicking the translation situation in plants. This is achieved by the viral kinase that is responsible for phosphorylating the RACK1 protein, leaving it at a similar level of phosphorylation and electronic charge to that found in the small ribosomal subunit of plants.

Other evidence is negative, in other words, it is based on the potential activities of viral tRNA-like structures that do not occur, which we have referred to previously: RNase P does not cut into the tRNA-like structures of HCV mRNA *in vivo* (Piron et al., 2005), and neither do tRNA-like structures of plant viruses act as amino acid donors in translation (Matsuda and Dreher, 2006, 2007). This reveals cellular functions that prevent the canonical expression of these signals, for example, the compartmentalization of RNase P in the cell nucleus. This suggests that, probably, the communicative potential of tRNA-like structures embedded in cellular mRNAs are also under cellular control so they do not express themselves, or they do so under very specific conditions, or in a very specific sense.

## Hypothetical Communicative Mechanism Involving tRNA-Like-mRNAs

It is well known that one of the elements involved in regulating the expression of mRNAs is mediated by microRNA binding to the mRNA, mainly in the 3′ UTR region, but also in the 5′ UTR region (Lytle et al., 2007). This binding usually inhibits the translation of cellular mRNA and can have various effects on viral mRNAs, including on their stability (Scheel et al., 2016). In recent years, a new form of communication has been revealed between mRNAs through these microRNAs. This communication is based on the existence of other RNAs that also have specific binding sites for microRNAs, including other long non-coding RNAs, circular and pseudogene RNAs, which compete with mRNAs in the binding of microRNAs affecting the translation rate of each of the messengers in the population. The wealth of microRNA signals and the topological ordering of their binding sites in messengers allow the existence of codes to be considered. This new way of communicating is known as competing endogenous RNAs (ceRNA) and has been denominated the “Rosetta Stone” of a hidden RNA language (Salmena et al., 2011; Tay et al., 2014). RNA viruses can also analogously participate in this type of communication. An example is the case of the microRNA specific to the human liver miR-122, which is necessary for the replication of the viral RNA. The binding of microRNA to viral RNA causes miR-122 sequestration from the cytoplasm (sponge effect) and alters the regulation of those cellular mRNAs on which miR-122 exerts its function (Luna et al., 2015). Recently, this type of communication has also been assigned to other microRNAs and a large group of diverse RNA viruses (Scheel et al., 2016).

Hypothetically, the communication mode of mRNAs carrying tRNA-like signals could be analogous to that of mRNAs with microRNA targets in ceRNA. On the one hand, the tRNA-like-mRNAs would act as specific targets for various cell factors that recognize tRNA, and on the other hand, the different forms related to tRNA (pre-tRNA, mature tRNA, tRNA-Aa, and fragments of mature tRNA (Gebetsberger et al., 2016) and SINE elements) would represent the role of competing RNAs in ceRNA communication. In the competition between tRNA-like-mRNA and the derivatives of tRNA for the factors that bond them, stoichiometry among competing RNAs is not the only important factor. The binding of the various factors to their target RNAs does not occur through simple Watson:Crick pairings, as in the case of microRNAs in the ceRNA-mediated communication model, but instead involves complex interactions. Thus, the differences between the affinity and kinetics constants of each of the factors for each of their target substrates in the mRNAs and that of their competitors, will play a much more important role in the case of the microRNA-mediated language, giving this type of communication a great deal of specificity. In addition, the factors would be capable of recognizing different sub-structures in the tRNA-like-mRNA [i.e., the T stem-loop and acceptor stem (Yuan and Altman, 1995), the elbow region (Zhang and Ferré-D’Amaré, 2016), etc.] and, as a function of this, different subgroups of cellular mRNAs carrying tRNA-like signals would be able to communicate coordinately. In a similar way to ceRNA-mediated communication, infection by a virus whose RNAs contains tRNA-like structures within them can sequester factors that normally bind the tRNA-like-mRNA signal. Releasing mRNA-tRNA-likes from these factors would allow these mRNAs to participate in another way in the communication and control relationships of the infected cell (i.e., enhanced or reduced stability and translation).

Although there is no experimental evidence of this entire process, there are results from parts of it that would provisionally support this hypothesis. One of these parts, that acting in the healthy cell, is represented by the example of negative self-regulation at the translational level of the expression of Threonyl-tRNA sinthetase (ThrS) in *E. coli* by the enzyme itself. Two tRNA-like structures in the mRNA leader sequence of the ThrS enzyme compete with tRNA^Thr^ for ThrS (Springer et al., 1989, 1998). An example of the other part, that of intersection between the biology of viral RNAs and tRNA is found HIV (Liu et al., 2016). The retrotranscription of the HIV-1 virus RNA needs tRNA^Lys^ as a primer for the reverse transcriptase enzyme. The virion contains copies of this tRNA to initiate the reproductive cycle in the newly infected cell, but this tRNA travels as a complex with the Lysyl-tRNA synthetase. So that this tRNA^Lys^ can be released from the complex, it needs the help of a tRNA-like element in the 5’ UTR region of the HIV mRNA, which competes with the interaction of that complex (Liu et al., 2016).

### Communication in a Social Context and Power Relations

The spread of a viral tRNA-like signal may change the context for the expression of tRNA-like-mRNAs. These changes could be related to a possible restoration of ancestral forms of cellular communication based on RNA signals that would have been silenced, repressed in the healthy cell (Gómez et al., 2015) and that the presence of the viral tRNA-like may release again. All this could extend the molecular “body” of the viral infection far beyond the mere production of more viruses. In any case, the biological interpretation of the tRNA-like-mRNA signals is an action in the conflict between the different agents in the cell, involving at least, antiviral mechanism on the one hand and cellular structures and functions that favor the infection on the other.

The concept of internal conflict and power, found in various branches of the humanities, has recently been adapted by several authors to accommodate molecular phenomena that do not fit clearly into the interpretation of the cell as a machine (Hurst et al., 1996; Gómez and Cacho, 2001; Cacho and Gómez, 2010). In some cases this takes the dialectic form, for example in the case of addiction systems (like the negation of an action that refers to the negation of another action), while in others it acquires more open forms, which reference Foucault’s philosophy of power (Cacho and Gómez, 2010) and can be applied to science without metaphysical contamination, and more generally as “actions on actions” (Garnar, 2006). Andrew Garnar summarizes: “power channels the directions in which meaning goes” (Garnar, 2006).

## Conclusion

The numerous structural elements embedded in cellular mRNA identified by RNase P could derive directly from proto-tRNA-like structures from the origin of life, or be indirectly related to these. In either case, these elements represent a record of primordial signals, a historical repository of memory brought to the present, not so much through the functions they originally represented (the “object” of the signal in the biosemiotic model (**Figure 1**), but by the potential of many archaic factors (RNase P, tRNA-aa-synthetase, ribosome subunits, etc.) to recognize and interpret these signals in the context of a present-day cell. Interaction or interpretive mediation does not have to be identical to that which occurred in a remote past, when these proto- tRNA-like signals were circulating as independent elements, but there is a possible analogy. We propose a competitive endogenous mechanism dependent on the tRNA-like structure (cetRNA) that would be compatible with primordial communication. This communicative web is the context that affects and is affected by the interpretation of the viral tRNA-like signal, but which is also modified by other signals or viral activities that repress or favor different connections in this network (Sharov et al., 2015).

Considering that the RNA virus may be originated back in the “RNA world,” that the tRNA-like structures are present in the virus infecting the three branches of life, and that tRNA-likes within mRNAs may have the same ancient origin, then a cell’s interpretation of both the viral tRNA-likes and its own tRNA-like-mRNAs during the infection, reveal the activity of a language at the structural RNA level that could ultimately refer to the “RNA world.” These tRNA-like structural elements embedded in cellular and viral mRNAs have been adaptive and not eliminated by live evolution, otherwise random drift and “neutral” synonymous mutations (Martínez et al., 2016). Trends in Microbiology would have easily disrupted these RNA structural motifs (Martínez et al., 2016). During an infection it could be possible to recover, at least partially, interactions from the remote past that have not been completely lost, but rather controlled. In this sense, the virus is a signal for the host cell (Gómez et al., 2015).

The importance of going experimentally deeper into this hypothesis involves verifying the simultaneous presence in the cell of historical layers of signaling and recognition. Viral tRNA-like structures could cross these layers and, simply through competition, displace factors that bind tRNA-like signals embedded in the messengers that would have been under control since the distant past, putting them in contact with present-day cell factors. In this sense, viruses represent a tool that allows submersion in the past through a non-phylogenetic method. It signifies direct immersion into other temporal layers where chronology is broken down, and it simultaneously establishes continuity between the viral mimetic signal and the context of the cellular interpretation of tRNA-like-mRNAs, whose agents refer to the origin of life. With this hypothesis in mind, it is possible that viruses can provide information on the possible molecular and consortial relationships of that primitive era.

## Author Contributions

The manuscript was written, discussed, edited, and reviewed by AA-M and JG.

## Conflict of Interest Statement

The authors declare that the research was conducted in the absence of any commercial or financial relationships that could be construed as a potential conflict of interest.
